# Associations of Urine Biomarkers During Ambulatory Acute Kidney Injury With Subsequent Recovery in Kidney Function: Findings From the SPRINT Study

**DOI:** 10.1053/j.ajkd.2025.02.607

**Published:** 2025-04-21

**Authors:** Simon B. Ascher, Ronit Katz, Michelle M. Estrella, Rebecca Scherzer, Teresa K. Chen, Pranav S. Garimella, Alexander L. Bullen, Stein I. Hallan, Nicholas Wettersten, Alfred Cheung, Michael G. Shlipak, Joachim H. Ix

**Affiliations:** Kidney Health Research Collaborative, Department of Medicine, San Francisco Veterans Affairs Health Care System and University of California San Francisco, San Francisco (SBA, MME, RS, TKC, MGS), Department of Internal Medicine, University of California, Davis, Sacramento (SBA), Division of Nephrology-Hypertension (PSG, ALB, JHI) and Division of Cardiology, Department of Medicine (NW), University of California, San Diego, La Jolla, Nephrology Section (ALB, JHI) and Cardiology Section (NW), Veterans Affairs San Diego Healthcare System, San Diego, California; Department of Obstetrics and Gynecology, University of Washington, Seattle, Washington (RK); Department of Clinical and Molecular Medicine, Faculty of Medicine, Norwegian University of Science and Technology (SIH), Department of Nephrology, St Olav University Hospital (SIH), Trondheim, Norway; and Division of Nephrology and Hypertension, University of Utah Health, Salt Lake City, Utah (AC)

## Abstract

**Rationale & Objective:**

Serum creatinine elevations in the ambulatory setting frequently occur during antihypertensive treatment and complicate clinical management, but few tools are available to distinguish whether kidney function will recover in this setting. This study evaluated whether urine biomarkers of glomerular and tubular health are associated with subsequent recovery of estimated glomerular filtration rate (eGFR) after acute kidney injury (AKI) has occurred in the ambulatory setting during blood pressure (BP) treatment.

**Study Design:**

Longitudinal analysis of clinical trial participants.

**Setting & Participants:**

652 participants in SPRINT (the Systolic Blood Pressure Intervention Trial) in whom AKI developed in the ambulatory setting, defined as an increase in serum creatinine of ≥0.3 mg/dL from baseline detected at the 1-year or 2-year study visit.

**Exposure:**

Eight urine biomarkers indexed to urine creatinine (Cr) measured at baseline and at the study visit when ambulatory AKI was detected.

**Outcome:**

<50% recovery in eGFR (“nonrecovery”) at 12 months.

**Analytical Approach:**

Multivariable logistic regression models, stratified by randomization arm, to evaluate biomarker associations with the odds of nonrecovery of eGFR.

**Results:**

Mean age was 70 ±10 years; eGFRs were 62 ± 25 mL/min/1.73 m^2^ at baseline and 42 ± 12 mL/min/1.73 m^2^ at the time of ambulatory AKI. Among biomarkers measured at the time ambulatory AKI was detected, higher urine albumin-Cr ratio (OR per 1–standard deviation higher, 1.72; 95% CI, 1.10–2.70) and lower epidermal growth factor/Cr (OR, 0.46; 95% CI, 0.26–0.79) were associated with nonrecovery of eGFR in the standard BP treatment arm; higher urine α-1 microglobulin-Cr ratio (OR, 1.45; 1.09–1.92), lower epidermal growth factor Cr ratio (OR, 0.62; 95% CI, 0.46–0.83), and lower kidney injury molecule–1-Cr ratio (OR, 0.75; 95% CI, 0.59–0.96) were associated with nonrecovery of eGFR in the intensive BP treatment arm.

**Limitations:**

Persons with diabetes and proteinuria >1 g/d were excluded.

**Conclusions:**

Among adults enrolled in a BP treatment trial who experienced ambulatory AKI, urine biomarkers reflecting glomerular injury and tubular dysfunction may help to distinguish whether kidney function will subsequently recover.

## PLAIN-LANGUAGE SUMMARY

Increases in serum creatinine concentration can occur when treating hypertension and complicate clinical management, but there are few tools available to distinguish whether an individual’s kidney function will subsequently recover. In this study, we investigated the association of kidney biomarkers measured in the urine with subsequent kidney function among individuals in the outpatient setting in whom an increase in serum creatinine occurs. We found that biomarkers reflecting worse glomerular injury and tubular dysfunction are associated with the risk of an individual’s kidney function not recovering. These results suggest that a broader assessment of kidney health when serum creatinine increases in the outpatient setting may help distinguish subsequent trajectories in kidney function.

Hypertension affects nearly half of all adults in the United States and is the leading modifiable contributor to cardiovascular disease (CVD) and early death.^1,2^ Blood pressure (BP) lowering confers substantial CVD and mortality benefits, but <25% of US adults with hypertension achieve a BP of <130/80 mm Hg.^3,4^ Acute kidney injury (AKI) occurs frequently in the ambulatory setting and is associated with hypertension and the use of renin-angiotensin-aldosterone system (RAAS) inhibitors and diuretic agents.^5,6^ Ambulatory AKI adds to the challenge of achieving BP control because clinicians may interpret increases in serum creatinine as a signal of intrinsic kidney damage and respond by deintensifying BP control or stopping medications such as RAAS inhibitors. However, recent studies show that increases in serum creatinine during intensive BP lowering often reflect benign hemodynamic changes.^7–9^ For these individuals, decisions to deintensify treatment may worsen CVD and chronic kidney disease (CKD) prognosis by reducing the time receiving optimal BP lowering therapy and cardio- and nephroprotective medications. Clinicians urgently need methods to distinguish hemodynamic perturbations from intrinsic kidney injury in ambulatory persons with AKI.

The kidney tubules comprise >90% of the kidney’s cortical structure, expend most of the energy used by the kidneys, and play a central role in a variety of essential kidney functions. Several studies show that urine biomarkers reflecting tubule function and injury are associated with multiple downstream adverse consequences in individuals with CKD, including AKI, kidney function decline, and CVD risk, beyond estimated glomerular filtration rate (eGFR) and albuminuria.^10–13^ Additional studies among adults hospitalized with AKI demonstrate that novel kidney tubule biomarkers measured in the inpatient setting or after hospital discharge are associated with adverse kidney outcomes,^14–17^ but, to our knowledge, there are limited data on whether kidney biomarkers measured at the time of an ambulatory AKI episode provide information about whether kidney function will subsequently recover.

In this ancillary study of SPRINT (Systolic Blood Pressure Intervention Trial) participants with ambulatory AKI, we measured eight urine biomarkers of glomerular injury and kidney tubule injury and dysfunction at baseline and at the time of ambulatory AKI and evaluated their associations with subsequent changes in eGFR. We hypothesized that biomarker concentrations indicating compromised kidney glomerular and tubular health at baseline and at the time of ambulatory AKI would be associated with subsequent nonrecovery in eGFR.

## Methods

### Study Design

The design and protocol of SPRINT have been reported previously.^18,19^ In brief, SPRINT was an open-label clinical trial that randomized participants with hypertension to an “intensive” systolic BP target of <120 mm Hg versus a “standard” BP target of <140 mm Hg. Inclusion criteria were age ≥50 years, systolic BP 130-180 mm Hg, and high CVD risk defined as prior clinical or subclinical CVD other than stroke, CKD (eGFR 20–59 mL/min/1.73 m^2^), age ≥75 years, or 10-year CVD risk >15% based on the Framingham Risk Score. Key exclusion criteria included diabetes mellitus, history of stroke, polycystic kidney disease, eGFR <20 mL/min/ 1.73 m^2^, and proteinuria >1 g/d. A total of 9,361 participants were randomized between November 2010 and March 2013 across 102 sites in the United States and Puerto Rico. The trial was stopped early after a median follow-up of 3.26 years on the advice of the data and safety monitoring board because of interim CVD results that favored the intensive BP lowering arm. The SPRINT study was approved by institutional review boards at each study site, and all participants provided written informed consent.

Among the 9,361 SPRINT participants, we identified 723 with or without CKD at baseline who (1) had ambulatory AKI, which was defined as a serum creatinine increase from baseline of ≥0.3 mg/dL that was detected at the 1-year or 2-year study visit (when urine specimens were collected and stored); and (2) did not experience one or more serious adverse events for AKI resulting in an emergency department visit or hospitalization during the follow-up period. Serum creatinine was measured at the SPRINT central laboratory (University of Minnesota, Minneapolis, MN) using an enzymatic creatinine method traceable to isotope dilute mass spectrometry (Roche). We then excluded 71 participants (9.8%) because of unavailable urine specimens, resulting in a final study sample of 652 participants. Eight urine biomarkers were measured at the baseline study visit and at the 1-year or 2-year study visit when the ambulatory AKI episode was detected. The urine biomarkers included measures reflecting glomerular injury (albumin), tubule reabsorptive function (α-1 microglobulin [α1m]), tubule synthetic function (epidermal growth factor [EGF] and uromodulin [UMOD]), and tubule injury and inflammation (interleukin-18 [IL-18], kidney injury molecule-1 [KIM-1], monocyte chemoattractant protein-1 [MCP-1], and chitinase-3-like protein-1 [YKL-40]).

This ancillary study was approved by the committees on human research at the University of California, San Francisco; the San Francisco Veterans Affairs Health Care System; and the Veterans Affairs San Diego Healthcare System. Patients and the public were not involved in the design or recruitment of this ancillary study or the dissemination of findings.

### Urine Biomarkers of Kidney Health

All urine specimens were processed immediately, shipped overnight on dry ice, and stored at −80°C until biomarker measurement without prior thaw. Urine albumin was measured by a nephelometric method using a ProSpec nephelometer (Siemens) at the SPRINT central laboratory. All other urine biomarkers were measured at the Kidney Health Research Collaborative Laboratory (San Francisco Veteran Affairs Health Care System, San Francisco, CA) by personnel blinded to clinical information. Biomarker analytic ranges, intraassay coefficients of variation, and assays are shown in Table S1. Urine biomarkers were measured in duplicate and averaged.

### Outcomes

The primary outcome of interest was a binary end point that categorized eGFR trajectories as recovery versus non-recovery at 12 months after the ambulatory AKI episode. The degree of eGFR change was calculated as follows:

(eGFR 12 months after AKI−eGFR at the time of AKI)/(eGFR at baseline−eGFR at the time of AKI×100)

This degree of change was then dichotomized as “recovery” (≥50%) versus “nonrecovery” (<50%). This definition was informed by guideline definitions for eGFR recovery following AKI in the inpatient setting.^20^ As a secondary outcome, we evaluated annualized percentage change in eGFR from the time of the ambulatory AKI episode to the end of the trial follow-up period, which was determined from a linear mixed-effects model based on serial serum creatinine measurements collected at 6-month intervals. Estimated GFR was calculated using the race-free 2021 CKD-EPI (Chronic Kidney Disease Epidemiology Collaboration) equation for creatinine.^21^

### Covariates

Age, sex, race, ethnicity, medical history, medications, and smoking status (current, former, or never) were obtained by questionnaire. Trained study coordinators measured BP using a standardized protocol and recorded BP as the mean of 3 seated measurements taken 1 minute apart after a 5-minute rest period using an automated oscillometric device (model 907; Omron Healthcare).^22^ Body mass index was calculated as weight in kilograms divided by height in meters squared. Covariate measurements at the 12-month or 24-month study visit were used.

### Statistical Analyses

**Figure 1.**
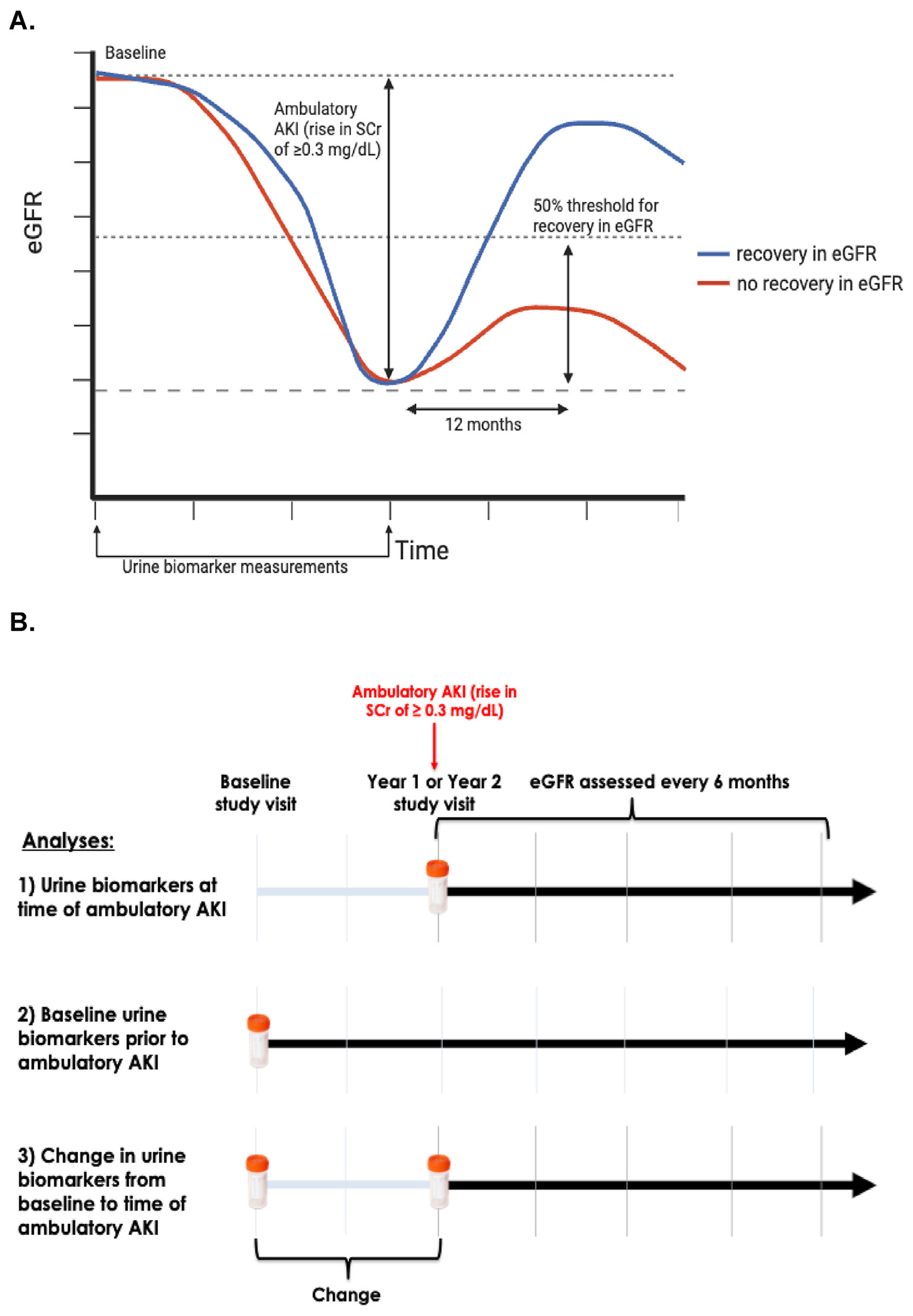
Study design evaluating associations of urine biomarkers of kidney health with change in estimated glomerular filtration rate (eGFR) following ambulatory acute kidney injury (AKI) in SPRINT (Systolic Blood Pressure Intervention Trial). (*A*) Study design for evaluating urine biomarker associations with the odds of eGFR nonrecovery at 12 months after ambulatory AKI. (*B*) Study design for evaluating urine biomarker associations with annualized change in eGFR after ambulatory AKI. Urine biomarkers were modeled as a single measurement at the time of ambulatory AKI, at the baseline trial visit, and as changes in urine biomarker concentrations from baseline to the time of ambulatory AKI. Abbreviation: Scr, serum creatinine.

For each urine biomarker, we modeled associations with nonrecovery in eGFR (<50%) at 12 months and annualized change in eGFR with 3 approaches: (1) using the urine biomarker concentration at the baseline visit of the trial (before the study visit when the ambulatory AKI event was detected), (2) using the urine biomarker concentration at the time of ambulatory AKI, and (3) using the relative change in urine biomarker concentration from the baseline study visit to the time of ambulatory AKI (Fig 1). Biomarkers were indexed to urine creatinine concentration to account for tonicity and then log-transformed and standardized to the same scale (mean, 0; SD, 1). Urine α1m had 14% of measurements below the lower limit of detection, compared with <2.5% of measurements of all other biomarkers; undetected values were imputed as the lower limit of detection divided by 2. Biomarker concentrations at the time of ambulatory AKI were modeled as continuous, log-linear predictors, and changes in biomarkers were analyzed in quartiles, with the lower 3 quartiles set as the reference group.

Logistic regression models were used to evaluate urine biomarker associations with odds of nonrecovery in eGFR. Linear mixed-effects models with random intercepts, random slopes, and an exchangeable covariance structure were used to evaluate urine biomarker associations with annualized change in eGFR. Fixed effects in the models included the urine biomarker concentration, time, and the interactions between the urine biomarker concentration and time, whereby the parameters of time and the interactions represent the annualized change in eGFR. The linear mixed-effects models used all available eGFR measurements for each participant; the median number of eGFR measurements after the serum creatinine increase was 5 (IQR, 4–6). To express annualized change in eGFR as a percentage, eGFR was natural log–transformed. All participants were followed until death or the last available follow-up when the trial was stopped in August 2015.

Analyses of biomarkers measured at baseline and at the time of ambulatory AKI were adjusted for covariates at the time of the biomarker measurement, and analyses of changes in biomarkers were adjusted for covariates at the baseline study visit. Adjustment variables included demographic characteristics (age, sex, race, and ethnicity), clinical characteristics (body mass index, smoking status, prevalent CVD, systolic BP and change in systolic BP from baseline, number of antihypertensive medications, and angiotensin-converting enzyme inhibitor or angiotensin receptor blocker use), eGFR, and urine albumin-creatinine ratio (ACR). The analyses of changes in biomarker levels were additionally adjusted for eGFR and urine ACR at the time of ambulatory AKI.

Interaction by randomization arm was evaluated in multivariable adjusted models using likelihood ratio tests. P values for interactions were adjusted for multiple testing by using the Benjamini-Hochberg procedure and setting the false discovery rate to 5%.^23^

In sensitivity analyses, we evaluated the associations of biomarkers measured at the time of ambulatory AKI with eGFR recovery at 6 months instead of 12 months. In addition, associations of biomarkers measured at the time of ambulatory AKI with annualized change in eGFR were evaluated in models that adjusted for urine creatinine concentration instead of indexing biomarkers to urine creatinine concentration and in models that censored eGFR follow-up at 2 years after the ambulatory AKI episode.

All analyses were conducted using Stata Statistical Software, release 13 (StataCorp LP) and SPSS Statistics for Windows, version 26.0 (IBM Corp).

## Results

Among the 652 SPRINT participants who experienced ambulatory AKI (increase in serum creatinine of ≥0.3 mg/dL) during follow-up and were included in this analysis, mean age at baseline was 70 ±10 years, 33% were women, and 72% were randomized to intensive BP lowering. Multiple significant interactions by randomized treatment assignment were identified; therefore, all analyses are reported stratified by randomization arm. The median increase in serum creatinine from baseline in both randomization arms was 0.4 mg/dL (IQR, 0.3–0.6). Among participants in the standard BP lowering arm (n = 180), the mean eGFRs at baseline and at the time of ambulatory AKI were 57 ± 26 mL/min/1.73 m^2^ and 38 ± 15 mL/min/1.73 m^2^, respectively. Among participants in the intensive BP lowering arm (n = 472), the mean eGFRs at baseline and at the time of ambulatory AKI were 64 ± 24 mL/min/1.73 m^2^ and 43 ± 14 mL/min/1.73 m^2^, respectively. Additional baseline characteristics stratified by treatment arm are shown in Table 1.

**Figure 2.**
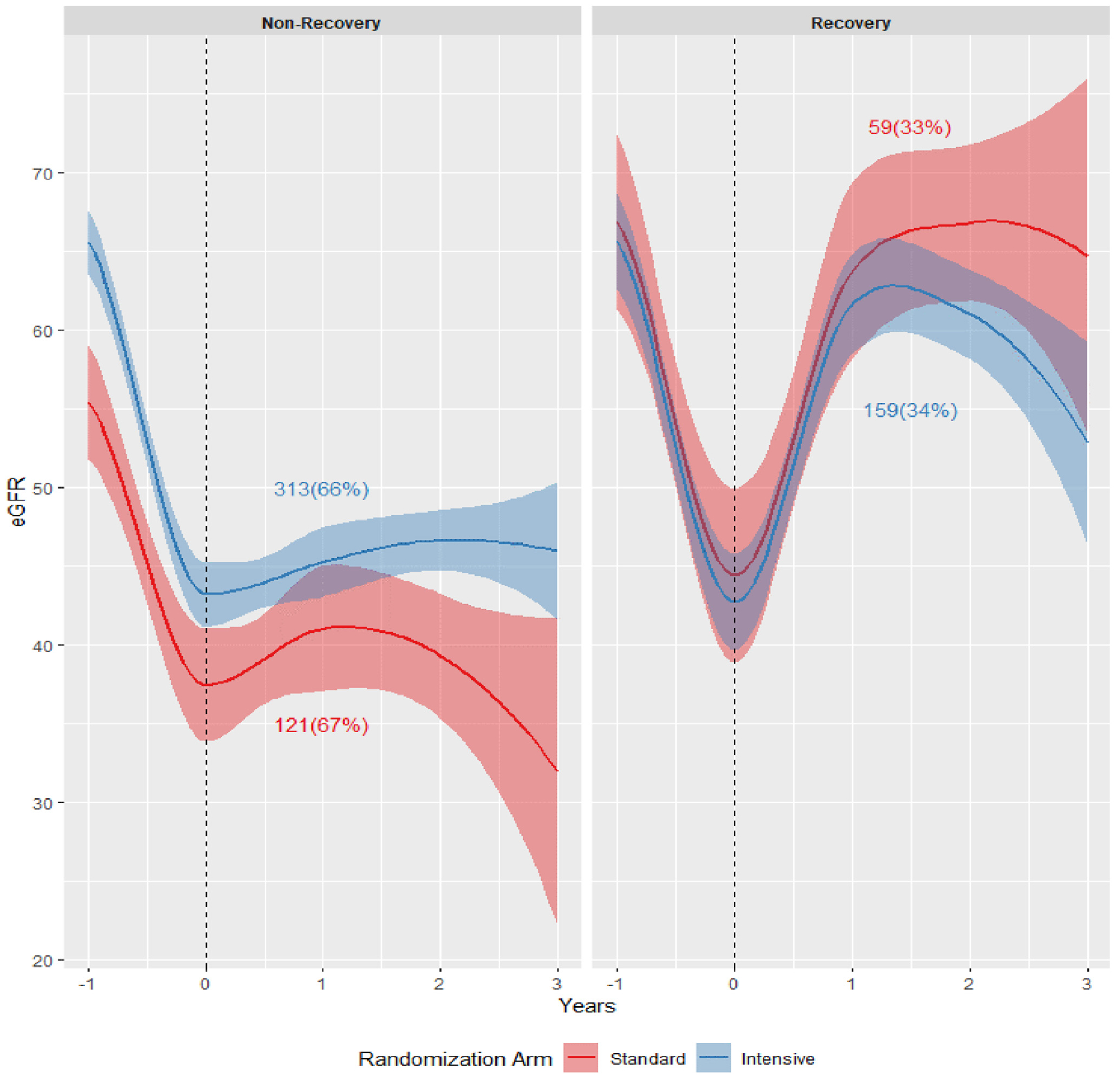
Mean estimated glomerular filtration rate (eGFR) after the time of ambulatory acute kidney injury stratified by eGFR recovery and randomized treatment assignment. Unadjusted mean eGFR from baseline to the end of follow-up with 95% CIs displayed. Results are stratified by randomized blood pressure treatment assignment and eGFR recovery versus nonrecovery. Numbers and percentages correspond to the proportion of participants in each randomized blood pressure treatment arm who experienced eGFR recovery or nonrecovery. Nonrecovery in eGFR is defined as an eGFR at 12 months after the ambulatory acute kidney injury episode recovering <50% of the loss in eGFR versus baseline.

Overall, 67% of participants (n = 434) experienced nonrecovery in eGFR at 12 months after the ambulatory AKI episode. Across treatment arms, there were similar proportions who experienced nonrecovery, and the eGFR trajectories among those with nonrecovery were similar (Fig 2). When evaluating biomarkers measured at the time of ambulatory AKI, higher urine ACR (odds ratio [OR] per 1-SD higher, 1.72; 95% CI, 1.10–2.70) and lower urine EGF-Cr ratio (OR, 0.46; 95% CI, 0.26–0.79) were associated with greater odds of subsequent nonrecovery of eGFR in the standard BP lowering arm. In the intensive BP lowering arm, a higher urine α1m/Cr and lower urine EGF/Cr and KIM-1/Cr were associated with greater odds of nonrecovery of eGFR (Table 2). A similar pattern of results was observed using baseline biomarker measurements and when assessing eGFR recovery at 6 months (Tables S2 and S3).

**Figure 3.**
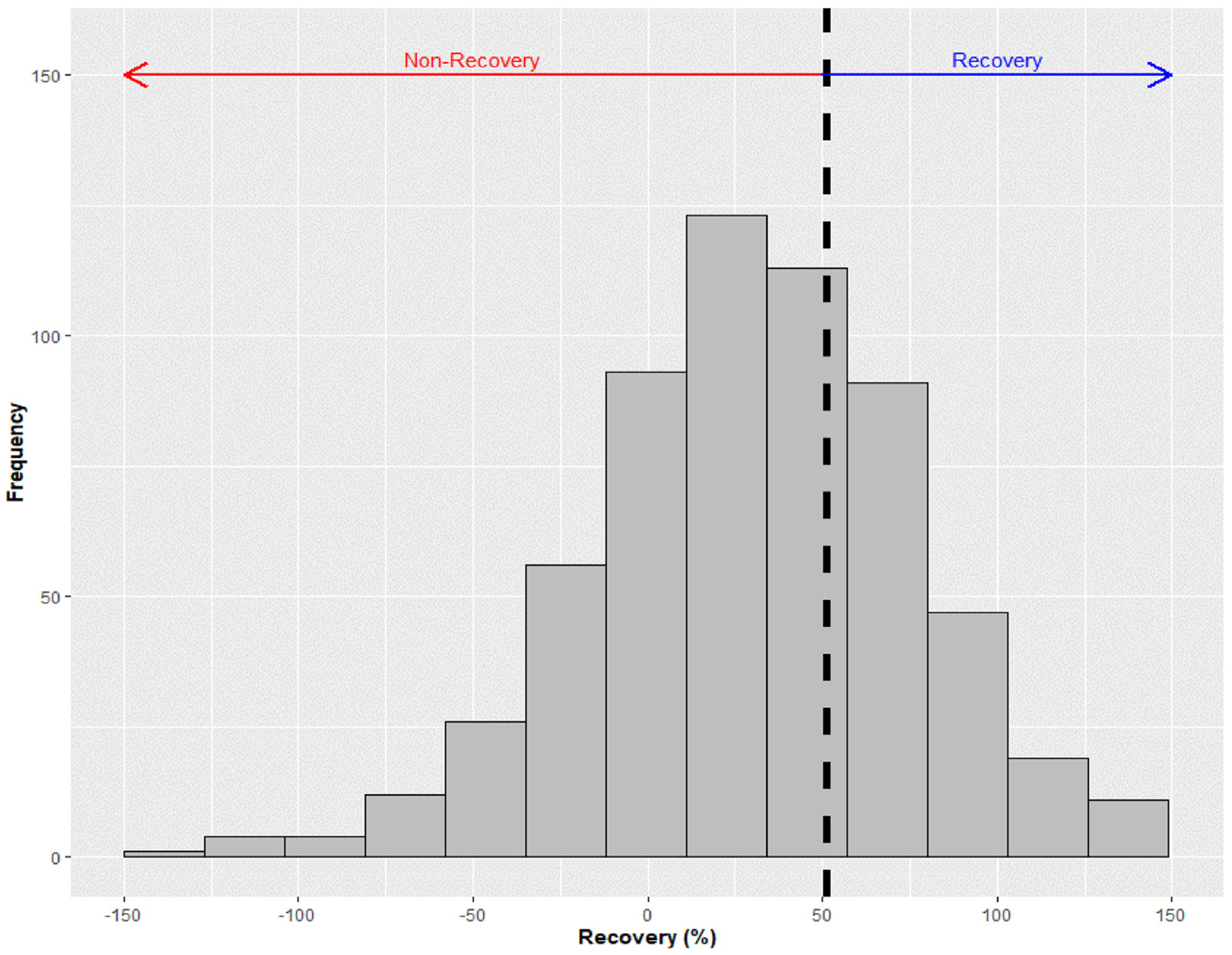
Distribution of estimated glomerular filtration rate (eGFR) recovery following ambulatory acute kidney injury in SPRINT (Systolic Blood Pressure Intervention Trial). Nonrecovery in eGFR is defined as an eGFR at 12 months after the ambulatory acute kidney injury episode recovering <50% of the loss in eGFR versus baseline.

The distribution of annualized change in eGFR after ambulatory AKI varied widely (Fig 3). In the standard BP lowering arm, mean annualized change in eGFR after ambulatory AKI was +3.8% per year (95% CI, 2.5–5.1). In the intensive BP lowering arm, mean annualized change in eGFR after ambulatory AKI was +4.4% per year (95% CI, 3.4–5.3). During a median of 2.0 years of follow-up, in the standard arm, higher urine ACR and lower urine EGF-Cr, UMOD-Cr, and MCP—1/Cr ratios were independently associated with faster subsequent declines in eGFR. These associations were generally similar in direction but weaker in the intensive BP lowering arm (all P < 0.05 for interaction; Table 3). Meanwhile, higher urine α1m-Cr ratio and lower urine KIM-1-Cr ratio at the time of ambulatory AKI were independently associated with a faster decline in eGFR in the intensive BP lowering arm, but not in the standard BP lowering arm. Results were similar using baseline biomarker measurements (Table S4). In sensitivity analyses, biomarker associations were also similar when they were adjusted rather than indexed to urine creatinine concentration and when annualized change in eGFR was censored at 2 years after ambulatory AKI (Table S5).

Percent changes in urine biomarker concentrations from baseline to the time of ambulatory AKI stratified by treatment arm and eGFR recovery are shown in Table S6. Most participants had increases in urine KIM-1 and MCP-1 concentrations and decreases in the other urine biomarkers, and the pattern of urine biomarker changes was largely similar in the standard and intensive BP lowering arms. Longitudinal changes in urine biomarkers were not associated with subsequent nonrecovery in eGFR in either treatment arm (Table S7).

## Discussion

In this ancillary study of SPRINT participants with and without CKD at baseline who experienced ambulatory AKI during follow-up, we observed that more extensive glomerular injury and tubular dysfunction assessed at baseline and at the time of the ambulatory AKI episode were independently associated with greater odds of nonrecovery in eGFR and faster annualized declines in eGFR. The associations were generally more pronounced among participants in the standard BP lowering arm than in the intensive BP lowering arm. These findings suggest that measuring urine biomarkers that reflect glomerular and kidney tubular dysfunction, but not tubular injury, at the time of ambulatory AKI may provide useful information about whether kidney function will subsequently recover.

To our knowledge, this is one of the first studies to evaluate kidney biomarker associations with eGFR recovery after ambulatory AKI; prior work has primarily focused on the potential role of kidney biomarkers in capturing the risk of adverse kidney outcomes after inpatient AKI.^14–17^ Our results are consistent with prior studies among SPRINT participants with CKD that showed that higher urine ɑ1m levels (reflecting worse proximal tubule function), lower urine UMOD levels (reflecting lower tubular synthetic capacity), and lower urine-to-plasma ratios of tubular secretion markers (reflecting lower tubular secretory capacity) are all associated with a greater risk of AKI episodes.^11,13,24^ Our findings are also supported by previous biomarker studies among adults with AKI in the inpatient setting, which demonstrated that higher urinary ACR and lower EGF and UMOD levels measured at baseline and at 3 months after an inpatient AKI episode are associated with greater risk of major adverse kidney events.^14–16^ Similarly, slower recovery in tubule function for as long as 12 months after an inpatient AKI episode was shown to be associated with a greater risk of CKD.^17^ Our study provides a novel extension of previous work by demonstrating among individuals with and without CKD that kidney biomarkers measured at the time of an ambulatory AKI episode may be helpful in distinguishing subsequent trajectories in kidney function.

Contrary to our hypotheses, we also observed that higher levels of urine KIM-1 and MCP-1 (reflecting worse proximal tubule injury) at the time of ambulatory AKI were associated with recovery rather than nonrecovery of kidney function, whereas other injury markers (urine IL-18 and YKL-40) had no associations. The absence of a consistent association across the injury markers with eGFR nonrecovery aligns with our prior work that demonstrated no associations between baseline tubular injury markers and clinical AKI events among SPRINT participants with CKD.^11^ These data together suggest that injury markers do not appear to be associated with the risk of AKI resulting in an emergency department visit or hospitalization or with recovery in eGFR following an ambulatory AKI episode. In contrast, other studies show that higher urinary MCP-1 and YKL-40 levels at the time of an inpatient AKI episode or at 3 or 12 months after discharge are associated with worse kidney function.^14,15,17^ The counterintuitive associations of several injury markers with recovery in eGFR after ambulatory AKI are difficult to explain and may be related to our nascent understanding of how to interpret ambulatory values of kidney biomarkers. Markers of tubule injury may be less specific than markers of tubule function in the ambulatory setting, as suggested by our prior work.^9^ In addition, the precise timing was unknown for each physiological event causing the ambulatory AKI episodes. If injury markers have different durations of increase after an ambulatory AKI episode relative to the tubule function measures, which was also suggested by our prior work,^9^ this could create a bias in their associations with AKI recovery. Alternatively, these may be chance findings. Additional studies are needed to reevaluate these associations and characterize the biological variation and longitudinal stability of ambulatory kidney biomarker measurements.

Serum creatinine increases commonly occur during hypertension treatment, but there are no tools available to distinguish whether an individual’s kidney function will subsequently recover. In our study, nearly half of the participants had subsequent improvements in eGFR after an ambulatory AKI episode, including one third who experienced a ≥50% recovery in eGFR. The varied eGFR trajectories following equivalent serum creatinine increases in our study underscore the difficulty clinicians face in making treatment decisions in this situation. Developing tools to help clinicians discern whether an increasing serum creatinine reflects intrinsic kidney injury or benign hemodynamic changes could have important implications for decision-making in regard to antihypertensive medications and BP targets for hypertension. We find that measuring biomarkers of kidney glomerular and tubular function at the time of an ambulatory AKI episode holds promise to inform the subsequent eGFR trajectory. We hypothesized that kidney tubule injury markers might also serve in this role, but higher injury biomarker levels were not consistently associated with changes in eGFR. If these findings are replicated in other settings, prospective studies would be warranted to determine whether biomarkers reflecting kidney glomerular and tubule function can guide the management of patients with ambulatory AKI.

The relatively stronger biomarker associations in the standard BP lowering arm compared with the intensive arm likely reflects the impact of BP lowering on urine biomarker concentrations. More intensive BP lowering causes hemodynamic changes to the glomerulus that may reduce the filtration of several urine biomarkers, particularly those that are freely filtered, such as α1m.^25^ The resulting reductions in urine biomarker concentrations may have attenuated associations with subsequent changes in eGFR among participants randomized to the intensive treatment arm, which would have decreased our study’s ability to distinguish biological signals for eGFR nonrecovery in the intensive BP lowering arm relative to the standard arm. Meanwhile, the proportion who experienced eGFR recovery after the ambulatory AKI event were similar in both BP treatment arms, suggesting that the utility of using novel markers to assess the potential cause and trajectory of eGFR recovery may have implications beyond those experiencing hemodynamic changes in the setting of intensive BP lowering.

We also observed that changes in biomarkers from baseline to the time of the ambulatory AKI had no associations with subsequent recovery of eGFR. We previously reported that SPRINT participants who experienced clinical AKI events had subsequently greater increases in markers reflecting worsening proximal tubular function and injury.^11^ In another study of persons living with HIV with preserved kidney function, longitudinal increases in kidney tubule biomarkers reflecting worsening proximal tubule function and tubular injury were associated with the risk of incident CKD.^26^ The findings in this study may relate to high inter- and intraindividual biologic variation in the biomarker concentrations, which is evident in the wide range of biomarker changes. We also anticipated that there would be changes in biomarker concentrations reflecting worsened glomerular and tubular health leading to the ambulatory AKI episode, but there was a general pattern of biomarker changes reflecting improved kidney health in both treatment arms despite developing ambulatory AKI. This suggests that most of the SPRINT participants included in our study may have experienced an ambulatory AKI episode because of benign hemodynamic changes.^7–9^ Although this pattern was more common in those in the intensive treatment arm, it was prevalent in the standard arm as well.

As an ancillary study of SPRINT, this analysis benefited from the use of protocol-driven eGFR assessments and urine collections, inclusion of individuals randomized to two different BP targets, and repeated biomarker measurements across multiple dimensions of kidney health. However, the study also has important limitations. First, although we were able to collect urine specimens at the same study visit as the ambulatory AKI episode, the extent to which the urine biomarker concentrations changed in response to the processes driving the serum creatinine elevation could not be completely captured. In addition, the timing and nature of the physiological event causing the ambulatory AKI episodes were unknown. Second, longitudinal prescription data were not available to determine whether medication changes after the ambulatory AKI episodes may have influenced eGFR recovery. Deescalation of antihypertensive medications may have led to short-term hemodynamic recovery in eGFR, but we anticipate this potential bias would apply equally to all participants regardless of their biomarker levels and should therefore influence our findings toward null results. Third, few CKD progression events accrued in SPRINT because it was designed as a CVD end point trial, excluded individuals with baseline eGFR <20 mL/min/1.73 m^2^ or severe proteinuria, and had a relatively short follow-up period. For this reason, we a priori selected nonrecovery of eGFR at 12 months after the ambulatory AKI episode as the primary end point for this analysis. Fourth, the magnitude of serum creatinine increases were similar, which prevented the present analysis from evaluating interaction by severity of AKI. Fifth, an external validation cohort was not available. Sixth, BP was measured in SPRINT using a standardized protocol that adheres to guideline recommendations, and standardized BP measurements are generally lower than BP measurements in routine clinical practice.^27–29^ Thus, our study may have had relatively more ambulatory AKI episodes that are hemodynamically mediated compared with the real-world clinical setting. Finally, our findings may not generalize to persons with heart failure, diabetes mellitus, or advanced CKD.

In summary, among individuals with hypertension without diabetes who experienced ambulatory AKI, urine biomarkers of glomerular injury and tubular dysfunction at the time of ambulatory AKI were associated with subsequent nonrecovery in kidney function, independent of eGFR, whereas biomarkers of tubule injury were not. Given the limitations of serum creatinine measurements in differentiating hemodynamic versus intrinsic causes of ambulatory AKI, these results strengthen the evidence that a broader assessment of kidney health can provide information about long-term kidney function trajectories and demonstrate the potential role of kidney biomarkers in guiding the clinical management of ambulatory AKI during hypertension treatment.

## Supplementary Material

1 **Table S1:** Biomarker analytic ranges, intraassay coefficients of variation, and assays
**Table S2:** Associations of baseline urinary biomarkers of kidney health measured 1 or 2 years before the ambulatory AKI event with subsequent recovery of eGFR
**Table S3:** Associations of urinary biomarkers of kidney health at the time of ambulatory AKI with subsequent recovery of eGFR at 6 months
**Table S4:** Associations of baseline urinary biomarkers of kidney health measured 1 or 2 years before the ambulatory AKI event with subsequent change in eGFR
**Table S5:** Sensitivity analyses of associations of urinary biomarkers of kidney health measured at the time of ambulatory AKI with subsequent change in eGFR
**Table S6:** Measures of kidney health at baseline and at the time of ambulatory AKI
**Table S7:** Associations of changes in urinary biomarkers of kidney health from baseline to the time of ambulatory serum creatinine increase with subsequent change in eGFR

## Figures and Tables

**Table 1.** Baseline Characteristics of SPRINT Participants With Ambulatory AKI

Characteristic	Standard BP (n = 180)	Intensive BP (n = 472)	Total (N = 652)
Age, y	70 ± 10	70 ± 9	70 ± 10
Female sex	68 (38%)	144 (31%)	212 (33%)
Race			
White	88 (49%)	264 (56%)	352 (54%)
Black	75 (42%)	155 (33%)	230 (35%)
Other	2 (1%)	9 (2%)	11 (2%)
Hispanic	15 (8%)	44 (9%)	59 (9%)
Smoking			
Never	71 (39%)	200 (42%)	271 (42%)
Former	72 (40%)	200 (42%)	272 (42%)
Current	37 (21%)	72 (15%)	109 (17%)
History of CVD	46 (26%)	119 (25%)	165 (25%)
History of heart failure	11 (6%)	22 (5%)	33 (5%)
Systolic BP, mm Hg	145 ± 17	144 ± 17	144 ± 17
Diastolic BP, mm Hg	77 ± 14	78 ± 13	78 ± 13
No. of antihypertensive medications	2.2 ± 1.1	1.9 ± 1.1	2.0 ± 1.1
Antihypertensive class			
β-Blocker	92 (51%)	201 (43%)	293 (45%)
Diuretic	85 (47%)	197 (42%)	282 (43%)
Calcium channel blocker	87 (48%)	204 (43%)	291 (45%)
ACEI or ARB	108 (60%)	254 (54%)	362 (56%)
BMI, kg/m^2^	29.3 ± 6.0	29.8 ± 2.3	29.6 ± 5.5
Serum creatinine, mg/dL	1.38 ± 0.57	1.22 ± 0.46	1.26 ± 0.50
eGFR, mL/min/1.73 m^2^	59 ± 25	66 ± 23	64 ± 24
Urine ACR, mg/g	26 (8–143)	15 (7–44)	16 (8–56)

Data presented as n (%), mean ± standard deviation, and median (interquartile range) where applicable. Abbreviations: ACEI, angiotensin-converting enzyme; ACR, albumin-creatinine ratio; AKI, acute kidney injury; ARB, angiotensin II receptor blocker; BMI, body mass index; BP, blood pressure; CVD, cardiovascular disease; eGFR, estimated glomerular filtration rate.

**Table 2.** Associations of Urinary Biomarkers of Kidney Health at Time of Ambulatory AKI With Subsequent Recovery in eGFR

Urine Biomarker	Standard (n = 180)		Intensive (n = 472)	
	Recovery^a^ (n = 59)	Nonrecovery^b^ (n = 121)	Recovery^a^ (n = 159)	Nonrecovery^b^ (n = 313)
**Glomerular injury**				
ACR	1.00 (ref)	1.68 (1.06–2.64)	1.00 (ref)	1.17 (0.88–1.55)

**Tubular reabsorption**				
α1m/Cr	1.00	1.05 (0.66–1.68)	1.00	1.45 (1.09–1.92)

**Synthetic function**				
EGF/Cr^c,d^	1.00	0.46 (0.27–0.80)	1.00	0.62 (0.46–0.83)

U MOD/Cr^c,d^	1.00	0.85 (0.58–1.26)	1.00	1.13 (0.91–1.41)

**Tubular injury**				
IL-18/Cr	1.00	1.23 (0.82–1.84)	1.00	1.02 (0.80–1.32)

KIM-1/Cr^c^	1.00	1.24 (0.82–1.87)	1.00	0.75 (0.59–0.97)

MCP-1/Cr	1.00	0.85 (0.53–1.38)	1.00	0.87 (0.70–1.09)

YKL-40/Cr	1.00	0.92 (0.61–1.37)	1.00	0.89 (0.70–1.14)

Data presented as OR (95% CI) per 1–standard deviation higher log-biomarker level. Models adjust for demographic characteristics (age, sex, and race/ethnicity), clinical risk factors (body mass index, smoking, history of cardiovascular disease, systolic blood pressure and change in systolic blood pressure from baseline, number of antihypertensive medications at baseline, angiotensin-converting enzyme inhibitor or angiotensin II receptor blocker use), baseline urine ACR, and baseline eGFR. Adjustment variables are measured at the time of urine biomarker measurement. Abbreviations: α1m, α-1 microglobulin; ACR, albumin-creatinine ratio; AKI, acute kidney injury; Cr, creatinine; EGF, epidermal growth factor; eGFR, estimated glomerular filtration rate; IL-18, interleukin-18; KIM-1, kidney injury molecule-1; MCP-1, monocyte chemoattractant protein-1; OR, odds ratio; UMOD, uromodulin; YKL-40, chitinase-3-like protein-1.

a Recovery in eGFR is defined as the eGFR 12 months after the time of Scr increase recovering ≥50% of the loss in eGFR versus baseline.

b Nonrecovery in eGFR is defined as the eGFR 12 months after the time of Scr increase recovering <50% of the loss in eGFR versus baseline.

c P < 0.05 for interaction by randomization arm.

d Higher urine EGR-CR ratio and UMOD-Cr ratio should be protective.

**Table 3.** Associations of Urinary Biomarkers of Kidney Health at Time of Ambulatory AKI With Subsequent Change in eGFR

Biomarker	Difference in Annualized eGFR Slope per 1-SD Higher Log-Biomarker Level, % per Year (95% CI)^a^	
	Standard (n = 180)	Intensive (n = 472)
**Glomerular injury**		
Urine ACR^b^	−4.68 (−5.82 to −3.54)	−1.79 (−2.84 to −0.74)

**Tubular reabsorption**		

Urine α1m/Cr	0.14 (−1.65 to 1.37)	−1.30 (−2.38 to −0.23)

**Synthetic function** ^c^		
Urine EGF/Cr^b^	4.71 (2.62 to 6.80)	2.06 (0.96 to 3.16)

Urine UMOD/Cr^b^	3.36 (1.80 to 4.92)	−0.79 (−1.63 to 0.06)

**Tubular injury**		
Urine IL-18/Cr	0.70 (−0.89 to 2.29)	−0.24 (−1.16 to 0.68)

Urine KIM-1/Cr^b^	−0.79 (−2.27 to 0.69)	1.38 (0.30 to 2.47)

Urine MCP-1/Cr	3.74 (1.84 to 5.64)	2.25 (1.34 to 3.17)

Urine YKL-40/Cr	0.67 (−0.87 to 2.21)	0.59 (−0.35 to 1.53)

Adjustment variables were measured at the time of urine biomarker measurement. Abbreviations: α1m, α-1 microglobulin; ACR, albumin-creatinine ratio; AKI, acute kidney injury; Cr, creatinine; EGF, epidermal growth factor; eGFR, estimated glomerular filtration rate; HR, hazard ratio; IL-18, interleukin-18; KIM-1, kidney injury molecule-1; MCP-1, monocyte chemoattractant protein-1; SD standard deviation; UMOD, uromodulin; YKL-40, chitinase-3-like protein-1.

a Models adjust for demographic characteristics (age, sex, and race/ethnicity), clinical risk factors (body mass index, smoking, history of cardiovascular disease, systolic blood pressure and change in systolic blood pressure from baseline, number of antihypertensive medications at baseline, angiotensin-converting enzyme inhibitor or angiotensin II receptor blocker use), and eGFR and urine ACR at the time of urine biomarker measurement.

b *P* < 0.05 for interaction by randomization arm.

c Higher urine EGF/Cr and UMOD/Cr should be protective.

## Data Availability

The data that support the findings of this study are available from the National Heart, Lung, and Blood Institute Biologic Specimen and Data Repositories and the corresponding author upon request.
