# Supplementary material for: Associations of Urine Biomarkers During Ambulatory Acute Kidney Injury With Subsequent Recovery in Kidney Function: Findings From the SPRINT Study
Source: Am J Kidney Dis. Author manuscript; Available in PMC 2026 Jun 3. (PMC13232060; doi:10.1053/j.ajkd.2025.02.607)
Supplement: 1 — Table S1: Biomarker analytic ranges, intraassay coefficients of variation, and assays Table S2: Associations of baseline urinary biomarkers of kidney health measured 1 or 2 years before the ambulatory AKI event with subsequent recovery of eGFR Table S3: Associations of urinary biomarkers of kidney health at the time of ambulatory AKI with subsequent recovery of eGFR at 6 months Table S4: Associations of baseline urinary biomarkers of kidney health measured 1 or 2 years before the ambulatory AKI event with subsequent change in eGFR Table S5: Sensitivity analyses of associations of urinary biomarkers of kidney health measured at the time of ambulatory AKI with subsequent change in eGFR Table S6: Measures of kidney health at baseline and at the time of ambulatory AKI Table S7: Associations of changes in urinary biomarkers of kidney health from baseline to the time of ambulatory serum creatinine increase with subsequent change in eGFR [file NIHMS2174847-supplement-1.pdf]

Table S1. Biomarker analytic ranges, intra-assay coefficients of variation, and assays

Biomarker	Analytic range	Intra-assay coefficient of variation	Biomarker assay
Urine α1m	5–480 mg/L	3.5–8.8%	Siemens nephelometric assay (Siemens, Tarrytown, NY)
Urine IL-18	2–10,000 ng/mL	4.9–13.7%	Multiplex assay (Meso Scale Diagnostics, Rockville, Maryland, USA)
Urine KIM-1	4–200,000 pg/mL	6.1–13.0%	Multiplex assay (Meso Scale Diagnostics, Rockville, Maryland, USA)
Urine MCP-1	3–10,000 pg/mL	7.1–12.0%	Multiplex assay (Meso Scale Diagnostics, Rockville, Maryland, USA)
Urine YKL-40	10–500,000 ng/mL	6.5–11.1%	Multiplex assay (Meso Scale Diagnostics, Rockville, Maryland, USA)
Urine UMOD	0.6–2510 ng/mL	11–19%	Multiplex assay (Meso Scale Diagnostics, Rockville, Maryland, USA)
Urine EGF	0–125,000 ng/mL	0–14.9%	Multiplex assay (Meso Scale Diagnostics, Rockville, Maryland, USA)

Abbreviations: α1m, alpha-1 microglobulin; EGF, epidermal growth factor; IL-18, interleukin-18; KIM-1, kidney injury molecule-1; MCP-1, monocyte chemoattractant protein-1; UMOD, uromodulin; YKL-40, chitinase-3-like protein-1.

Table S2. Associations of baseline urinary biomarkers of kidney health measured one or two years prior to the ambulatory AKI event with subsequent recovery in eGFR

	OR (95% CI) per 1-SD higher log-biomarker level			
	**Standard arm (n=180)**		**Intensive arm (n=472)**	
**Urine biomarker**	**Recovery[a] (n=60)**	**Non-recovery[b] (n=120)**	**Recovery[a] (n=175)**	**Non-recovery[b] (n=297)**
Glomerular injury				
Urine ACR	1.00 (Ref)	1.36 (0.93, 1.99)	1.00 (Ref)	**1.32 (1.03, 1.68)**
Tubular reabsorption				
Urine α1m/Cr	1.00	1.37 (0.91, 2.07)	1.00	1.27 (0.99, 1.61)
Synthetic function				
Urine EGF/Cr[c,d]	1.00	**0.36 (0.19, 0.68)**	1.00	0.77 (0.56, 1.05)
Urine UMOD/Cr[c,d]	1.00	0.95 (0.67, 1.34)	1.00	1.10 (0.89, 1.36)
Tubular injury				
Urine IL-18/Cr	1.00	1.21 (0.81, 1.80)	1.00	0.94 (0.75, 1.18)
Urine KIM-1/Cr[c]	1.00	1.07 (0.73, 1.56)	1.00	**0.77 (0.61, 0.97)**
Urine MCP-1/Cr	1.00	1.15 (0.80, 1.66)	1.00	1.01 (0.82, 1.25)
Urine YKL-40/Cr	1.00	1.12 (0.81, 1.54)	1.00	0.96 (0.76, 1.22)

Abbreviations: α1m, alpha-1 microglobulin; ACR, albumin-to-creatinine ratio; AKI, acute kidney injury; Cr, creatinine; EGF, epidermal growth factor; eGFR, estimated glomerular filtration rate; IL-18, interleukin-18; KIM-1, kidney injury molecule-1; MCP-1, monocyte chemoattractant protein-1; OR, odds ratio; UMOD, uromodulin; YKL-40, chitinase-3-like protein-1.

[a] Recovery in eGFR defined as eGFR 12 months after the ambulatory AKI episode recovering ≥50% of the loss in eGFR from baseline.

[b] Non-recovery in eGFR defined as eGFR 12 months after the ambulatory AKI episode recovering <50% of the loss in eGFR from baseline.

[c] P for interaction by randomization arm <0.05.

[d] Higher urine EGF/Cr and UMOD/Cr should be protective.

Table S3. Associations of urinary biomarkers of kidney health at the time of ambulatory AKI with subsequent recovery in eGFR at 6 months

	OR (95% CI) per 1-SD higher log-biomarker level			
	Standard arm (n=180)		Intensive arm (n=472)	
Urine biomarker	Recovery[a] (n=60)	Non-recovery[b] (n=120)	Recovery[a] (n=175)	Non-recovery[b] (n=297)
Glomerular injury				
Urine ACR	1.00 (Ref)	**1.56 (1.03, 2.37)**	1.00 (Ref)	1.19 (0.90, 1.58)
Tubular reabsorption				
Urine α1m/Cr	1.00	1.06 (0.68, 1.68)	1.00	**1.30 (1.01, 1.68)**
Synthetic function				
Urine EGF/Cr[c,d]	1.00	**0.52 (0.31, 0.89)**	1.00	**0.73 (0.55, 0.98)**
Urine UMOD/Cr[c,d]	1.00	1.05 (0.71, 1.54)	1.00	0.99 (0.92, 1.32)
Tubular injury				
Urine IL-18/Cr	1.00	1.17 (0.80, 1.72)	1.00	0.90 (0.71, 1.16)
Urine KIM-1/Cr[c]	1.00	1.07 (0.72, 1.59)	1.00	**0.67 (0.52, 0.86)**
Urine MCP-1/Cr	1.00	0.87 (0.54, 1.41)	1.00	**0.77 (0.61, 0.96)**
Urine YKL-40/Cr	1.00	1.17 (0.79, 1.73)	1.00	0.83 (0.65, 1.06)

Abbreviations: α1m, alpha-1 microglobulin; ACR, albumin-to-creatinine ratio; AKI, acute kidney injury; Cr, creatinine; EGF, epidermal growth factor; eGFR, estimated glomerular filtration rate; IL-18, interleukin-18; KIM-1, kidney injury molecule-1; MCP-1, monocyte chemoattractant protein-1; OR, odds ratio; UMOD, uromodulin; YKL-40, chitinase-3-like protein-1.

Note: Models adjust for demographics (age, sex, and race/ethnicity), clinical risk factors (body mass index, smoking, history of cardiovascular disease, systolic blood pressure, number of antihypertensive medications at baseline, angiotensin converting enzyme inhibitor or angiotensin II receptor blocker use), baseline urine ACR, and baseline eGFR. Adjustment variables measured at time of urine biomarker measurement.

[a] Recovery in eGFR defined as eGFR 12 months after the ambulatory AKI episode recovering ≥50% of the loss in eGFR from baseline.

[b] Non-recovery in eGFR defined as eGFR 12 months after the ambulatory AKI episode recovering <50% of the loss in eGFR from baseline.

[c] P for interaction by randomization arm <0.05.

[d] Higher urine EGF/Cr and UMOD/Cr should be protective.

Table S4. Associations of baseline urinary biomarkers of kidney health measured one or two years prior to the ambulatory AKI event with subsequent change in eGFR

	Difference in annualized eGFR slope per 1-SD higher log-biomarker level (% per year) (95% CI)	
**Urine biomarker**	**Standard arm (n=180)**	**Intensive arm (n=472)**
Glomerular injury		
Urine ACR[b]	**-4.21 (-5.56, -2.87)**	**-1.80 (-2.69, -0.90)**
Tubular reabsorption		
Urine α1m/Cr	**-2.14 (-3.54, -0.73)**	-0.60 (-1.62, 0.42)
Synthetic function[a]		
Urine EGF/Cr	**5.85 (3.86, 7.84)**	**1.60 (0.60, 2.61)**
Urine UMOD/Cr	-0.48 (-2.19, 1.22)	-0.29 (-1.15, 0.57)
Tubular injury		
Urine IL-18/Cr	0.59 (-1.16, 2.34)	**0.95 (0.06, 1.84)**
Urine KIM-1/Cr	0.71 (-0.92, 2.34)	0.87 (-0.11, 1.85)
Urine MCP-1/Cr	-0.34 (-2.00, 1.32)	0.82 (-0.11, 1.75)
Urine YKL-40/Cr	0.07 (-1.29, 1.43)	**1.03 (0.11, 1.96)**

Abbreviations: α1m, alpha-1 microglobulin; ACR, albumin-to-creatinine ratio; AKI, acute kidney injury; CI confidence interval, Cr, creatinine; EGF, epidermal growth factor; eGFR, estimated glomerular filtration rate; HR, hazard ratio; IL-18, interleukin-18; KIM-1, kidney injury molecule-1; MCP-1, monocyte chemoattractant protein-1; SD standard deviation; UMOD, uromodulin; YKL-40, chitinase-3-like protein-1.
Note: Models adjusted demographics (age, sex, and race/ethnicity), clinical risk factors (body mass index, smoking, history of cardiovascular disease, systolic blood pressure, number of antihypertensive medications at baseline, angiotensin converting enzyme inhibitor or angiotensin II receptor blocker use), and eGFR and urine ACR at time of urine biomarker measurement. Adjustment variables measured at time of urine biomarker measurement.
[a] Higher urine EGF/Cr and UMOD/Cr should be protective.

Table S5. Sensitivity analyses of associations of urinary biomarkers of kidney health at the time of ambulatory AKI with subsequent change in eGFR

	Difference in annualized eGFR slope per 1-SD higher log-biomarker level (% per year) (95% CI)		
**Urine biomarker**	**Original analysis**	**Adjusting instead of indexing for UCr**	**Censoring eGFR at 2 years follow-up**
**Standard arm (n=180)**			
Glomerular injury			
Urine ACR	**-4.49 (-5.59, -3.38)**	**-5.24 (-6.46, -4.03)**	**-5.05 (-6.79, -3.31)**
Tubular reabsorption			
Urine α1m/Cr	0.01 (-1.44, 1.46)	-1.57 (-3.18, 0.05)	0.41 (-1.98, 2.79)
Synthetic function[a]			
Urine EGF/Cr	**4.25 (2.28, 6.22)**	**5.82 (2.72, 8.92)**	**3.97 (1.37, 6.57)**
Urine UMOD/Cr	**3.42 (1.88, 4.96)**	**1.99 (0.29, 3.69)**	**2.64 (0.42, 4.86)**
Tubular injury			
Urine IL-18/Cr	0.78 (-0.79, 2.34)	1.16 (-0.92, 3.24)	0.97 (-1.31, 3.25)
Urine KIM-1/Cr	-0.67 (-2.12, 0.78)	1.91 (-0.55, 4.36)	-0.92 (-3.13, 1.29)
Urine MCP-1/Cr	**3.83 (1.97, 5.70)**	**5.34 (2.77, 7.93)**	**4.73 (1.97, 7.49)**
Urine YKL-40/Cr	0.78 (-0.73, 2.29)	1.19 (-0.55, 2.93)	0.82 (1.41, 3.06)
**Intensive arm (n=472)**			
Glomerular injury			
Urine ACR	**-1.74 (-2.79, -0.68)**	**-1.50 (-2.58, -0.42)**	**-2.10 (-3.51, -0.68)**
Tubular reabsorption			
Urine α1m/Cr	**-1.33 (-2.40, -0.26)**	-0.86 (-1.91, 0.19)	**-2.03 (-3.45, -0.61)**
Synthetic function[a]			
Urine EGF/Cr	**2.06 (0.96, 3.16)**	**2.94 (1.25, 4.63)**	**2.86 (1.42, 4.30)**
Urine UMOD/Cr	-0.79 (-1.64, 0.06)	-0.28 (-1.24, 0.67)	-0.30 (-1.46, 0.86)
Tubular injury			
Urine IL-18/Cr	-0.34 (-1.26, 0.58)	-0.54 (-1.69, 0.60)	-0.37 (-1.65, 0.90)
Urine KIM-1/Cr	**1.41 (0.32, 2.51)**	**1.56 (-0.03, 3.15)**	**2.01 (0.58, 3.45)**
Urine MCP-1/Cr	**2.30 (1.38, 3.22)**	**2.77 (1.58, 3.96)**	**2.70 (1.54, 3.86)**
Urine YKL-40/Cr	0.52 (-0.42, 1.47)	0.56 (-0.46, 1.57)	0.88 (-0.44, 2.19)

Abbreviations: α1m, alpha-1 microglobulin; ACR, albumin-to-creatinine ratio; AKI, acute kidney injury; CI confidence interval, Cr, creatinine; EGF, epidermal growth factor; eGFR, estimated glomerular filtration rate; HR, hazard ratio; IL-18, interleukin-18; KIM-1, kidney injury molecule-1; MCP-1, monocyte chemoattractant protein-1; SD standard deviation; UMOD, uromodulin; YKL-40, chitinase-3-like protein-1.

Note: Models adjusted demographics (age, sex, and race/ethnicity), clinical risk factors (body mass index, smoking, history of cardiovascular disease, systolic blood pressure, number of antihypertensive medications at baseline, angiotensin converting enzyme inhibitor or angiotensin

II receptor blocker use), and eGFR and urine ACR at time of urine biomarker measurement. Adjustment variables measured at time of urine biomarker measurement.
[a] Higher urine EGF/Cr and UMOD/Cr should be protective.

Table S6. Measures of kidney health at baseline and at the time of ambulatory AKI

	Standard arm (n=180)			Intensive arm (n=472)		
**Measure of kidney health**	**Baseline**	**Time of SCr elevation**	**Percentage change (%)**	**Baseline**	**Time of SCr elevation**	**Percentage change (%)**
eGFR	59 (25)	40 (15)	-31 (10)	66 (24)	43 (14)	-33 (9)
Urine ACR, mg/g	26 [8, 143]	18 [8, 123]	-13 [-54, 65]	15 [7, 44]	9 [5, 23]	-38 [-67, 8]
Urine α1m, mg/L	14 [8, 30]	16 [7, 30]	-2 [-44, 83]	11 [7, 20]	10 [5,19]	-16 [-44, 40]
Urine EGF, pg/L	29 [17,45]	20 [10,31]	-27 [-42,-11]	35 [22,51]	22 [15,31]	-33 [-49, -16]
Urine UMOD, mg/mL	37 [23, 56]	27 [15,43]	-22[-58, 24]	39 [25, 65]	28 [14, 48]	-30 [-58, 10]
Urine IL-18, pg/mL	43 [24, 69]	37 [21, 60]	-17 [-47, 50]	37 [24, 60]	29 [18, 50]	-23 [-48, 21]
Urine KIM-1, pg/mL	480 [236,805]	600 [204, 1091]	16 [-25, 91]	441 [260,753]	533 [300, 928]	14 [-25, 66]
Urine MCP-1, pg/mL	189 [130, 280]	299 [170, 436]	42 [-4, 122]	181 [127, 262]	261 [163,462]	42 [-8, 149]
Urine YKL-40, pg/mL	548 [296, 1108]	544 [170,1410]	-5 [-64, 136]	534 [293,1056]	385 [193,935]	-30 [-63,50]
Urine creatinine	111 [69, 161]	118 [74, 168]	12 [-26, 62]	113 [76, 163]	131 [86, 185]	13 [-21, 71]

Abbreviations: α1m, alpha-1 microglobulin; ACR, albumin-to-creatinine ratio; AKI, acute kidney injury; EGF, epidermal growth factor; eGFR, estimated glomerular filtration rate; IL-18, interleukin-18; KIM-1, kidney injury molecule-1; MCP-1, monocyte chemoattractant protein-1; SCr, serum creatinine; UMOD, uromodulin; YKL-40, chitinase-3-like protein-1. Data presented as mean (standard deviation) or median [interquartile range].

Table S7. Associations of changes in urinary biomarkers of kidney health from baseline to the time of ambulatory AKI with subsequent eGFR recovery

	OR (95% CI) for eGFR recovery between highest quartile versus lower three quartiles of biomarker change[a]			
	Standard arm (n=180)		Intensive arm (n=472)	
Urine biomarker[b]	Q1-Q3 biomarker change	Q4 biomarker change	Q1-Q3 biomarker change	Q4 biomarker change
Glomerular injury				
Urine ACR	1.00 (Ref)	1.76 (0.75, 4.08)	1.00 (Ref)	0.95 (0.57, 1.58)
Tubular reabsorption				
Urine α1m/Cr	1.00	1.58 (0.68, 3.64)	1.00	1.36 (0.78, 2.36)
Synthetic function[c]				
Urine EGF/Cr	1.00	0.94 (0.42, 2.08)	1.00	0.75 (0.45, 1.22)
Urine UMOD/Cr	1.00	0.72 (0.32, 1.62)	1.00	0.90 (0.54, 1.50)
Tubular injury				
Urine IL-18/Cr	1.00	1.97 (0.83, 4.69)	1.00	1.18 (0.69, 1.99)
Urine KIM-1/Cr	1.00	0.48 (0.20, 1.16)	1.00	1.23 (0.75, 2.02)
Urine MCP-1/Cr	1.00	0.78 (0.31, 1.95)	1.00	0.93 (0.58, 1.50)
Urine YKL-40/Cr	1.00	0.86 (0.37, 1.99)	1.00	0.76 (0.45, 1.29)

Abbreviations: α1m, alpha-1 microglobulin; ACR, albumin-to-creatinine ratio; AKI, acute kidney injury; Cr, creatinine; EGF, epidermal growth factor; eGFR, estimated glomerular filtration rate; IL-18, interleukin-18; KIM-1, kidney injury molecule-1; MCP-1, monocyte chemoattractant protein-1; OR, odds ratio; UMOD, uromodulin; YKL-40, chitinase-3-like protein-1.

Note: Models adjust for demographics (age, sex, and race/ethnicity), clinical risk factors (body mass index, smoking, history of cardiovascular disease, systolic blood pressure, number of antihypertensive medications at baseline, angiotensin converting enzyme inhibitor or angiotensin II receptor blocker use), baseline urine ACR, and baseline eGFR. Adjustment variables measured at time of the serum creatinine elevation.

[a] Recovery in eGFR defined as eGFR 12 months after time of serum creatinine elevation recovering ≥50% of the loss in eGFR from baseline.

[b] P for interaction by randomization arm >0.10 for all biomarkers.

[c] Greater increases in urine EGF/Cr and UMOD/Cr should be protective.
